# Functional implications of radical neck dissection and the impact on the quality of life for patients with head and neck neoplasia

**Published:** 2012-12-25

**Authors:** B Popescu, SVG Berteșteanu, R Grigore, R Scăunașu, CR Popescu

**Affiliations:** * E.N.T. Department, Coltea Clinical Hospital, Bucharest, Romania; ** General Surgery Department, Colțea Clinical Hospital, Bucharest, Romania

**Keywords:** nutritional status, routine surgical procedure, deformities

## Abstract

Radical neck dissection is a concept that was presented in 1906 by GW Crile and suffered constant improvement ever since. The surgical oncology procedure included the resection of the internal jugular vein, the sternocleidomastoid muscle, and the submandibular gland and the spinal accessory nerve. Deformities and impairment in the functionality of different regions of the neck and scapular regions have great implications on the quality of life of the patients who undergo such a procedure. Modifications to the radical neck dissection were made in the attempt to maintain the efficacy of the surgical oncology therapy. The authors try to assess the functional implications of radical neck dissection and the impact on the quality of life for patients with head and neck neoplasia.

## Introduction

In 1906, GW Crile was the first physician who described the radical neck dissection in patients with malignant neoplasia in the head and neck region. The technique encompasses the need of surgical removal of the tumor metastasis located in the lymphatic structures from the mandible to the clavicle and from the trapezius muscle in the posterior to the median region of the anterior neck along with anatomical structures that may be invaded by the malignant tumor. The surgical oncology procedure included the resection of the internal jugular vein, the sternocleidomastoid muscle, and the submandibular gland and the spinal accessory nerve. In terms of oncology prospects, this is a routine surgical procedure performed for N3 metastasis and for extensive tumor growth. 

Deformities and impairment in the functionality of different regions of the neck and scapular regions have great implications on the quality of life of the patients who undergo such a procedure. Taking this into consideration E. Bocca and O. Suarez independently described an alternative for the radical neck dissection that involved the removal of the entire lymph tissue in the neck but conserving one or more anatomic structures without the sacrifice of the oncological efficacy. This led to decreased deformities and a better quality of life for the head and neck cancer patients.

## Method

Head and neck cancer patients who are submitted in the E.N.T. Department of Coltea Clinical Hospital have a tendency of presenting to the specialist in late stages of the tumor process thus needing an extensive surgical procedure. Most of the patients are male (85%) who have a low social status, are heavy smokers, alcohol drinkers and have an altered nutritional status. More than 75% of the patients have tumor induced cachexia which in terms of outcome is a poor prognostic factor.

 The diagnostic procedures include clinical exam and imaging studies. This allows us to assess the extent of the tumor process so that a correct surgical indication can be drawn. Imaging studies include ultrasonography, CT scans, plane X-ray investigation, magnetic resonance investigation, and positron emission tomography. The nutritional status of the patients is calculated according to the ESPEN guidelines by using the nutritional risk score NRS 2002 [**1**]. After participating in the Nutrition Day surveys from 2008 to 2010 we came to the conclusion that head and neck cancer patients who have an altered nutritional status need to be stabilized by using enteral nutrition, if the digestive system is not impaired, parenteral nutrition or mixed nutrition. A well-fed patient has a better prognostic because of the increased activity of the immune system, a more rapid wound healing process, shorter hospitalization period with the potential of a more rapid social reintegration. 

 In 1991, the American Academy of Otolaryngology-Head and Neck Surgery (AAO-HNS) published an official report that issued a standardized terminology for the different types of neck dissection [**2**]. This classification was necessary in order to optimize the neck dissections that were performed up until that time and to clarify the surgical indications for the procedure [**3**]. This report was improved in 2001 when the AAO-HNS presented an update for this classification in which the presence of 6 lymph node levels with 6 sublevels and an additional 7th level which is present in the upper mediastinum were stated [**4**]. This classification is also used in our clinic.

There is an alternative for the radical neck dissection in terms of conserving anatomical structures if the tumor process invasion allows the use of a less invasive surgical procedure [**5**]. Modifications to the radical neck dissection were made in the attempt to maintain the efficacy of the surgical oncology therapy and include the following types:

 • Type I: The preservation of the accessory spinal nerve.

 • Type II: The preservation of the accessory spinal nerve and the internal jugular vein. 

 • Type III: The preservation of the accessory spinal nerve, the internal jugular vein and the stranocleidomastoid muscle.

**Fig. 1:**
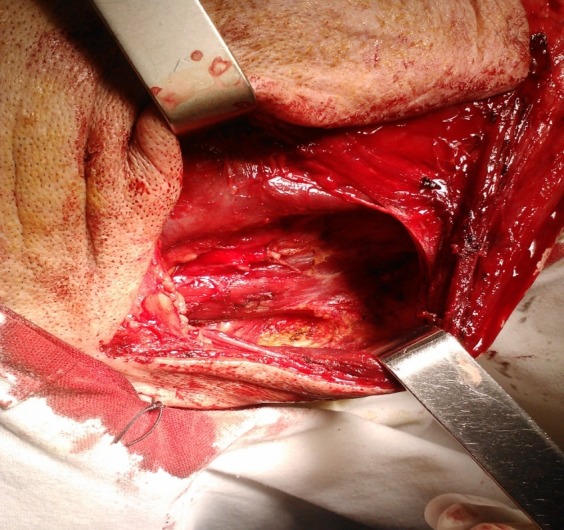
Resected spinal accessory nerve. Modified neck dissection

 When sacrificing the spinal accessory nerve the patient will experience impairment in the movement of the shoulder with the fixation of the shoulder and with or without pain sensations in the territory of the cranial nerve, which reside in the denervation of the trapezius muscle (**Fig. 1**). When performing type I to III modified radical neck dissections, the patient will not experience any impairment in moving the arm and shoulder, which, in terms of function and social reintegration will give the patient a start point [**6**]. Pain resulting from the resection of the spinal accessory nerve is subject to the administration of long-term anti-pain drugs. This is a positive aspect of the surgical therapy because the patient has a better adherence to the oncological therapy. 

**Fig. 2:**
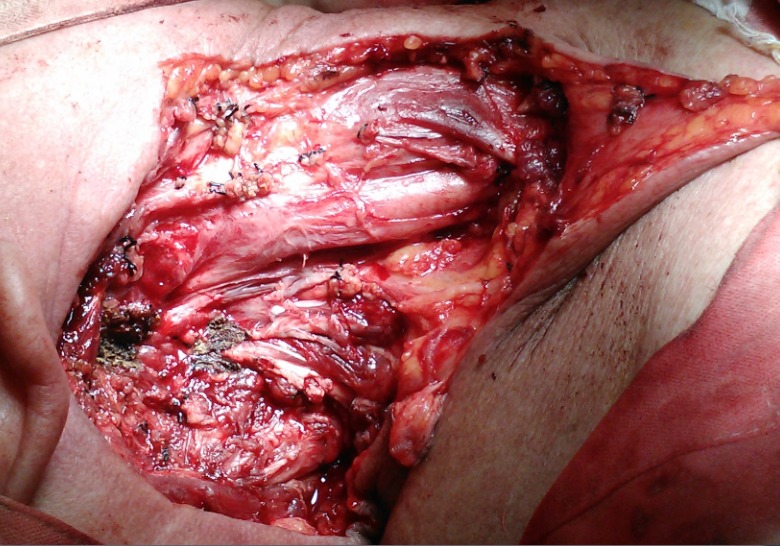
Radical neck dissection. Exposed carotid arteries

 The removal of the sternocleidomastoid muscle has a great impact on the esthetic aspect of the patient because of the deformity it creates in the cervical region. This also includes the flattening of the neck in the lateral region of the neck [**7**]. Patients accuse a certain social deferral when having to deal with such situations. The functional deficit for these patients is minimal, in some even absent. Cervical vessels are protected by the presence of the sternocleidomastoid muscle. When resecting this muscle, the vessels are only protected by the superficial layers of the anatomical structures (platysma muscle, superficial layers of the neck fascia, fatty tissue and skin) and, in this resides the need of the patient to protect that region in the neck (**Fig. 2**). 

**Fig. 3:**
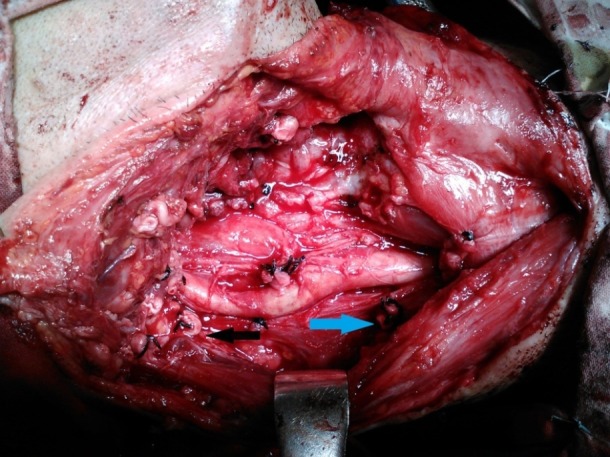
Radical neck dissection. Black arrow indicates the resected internal jugular vein. Blue arrow indicates the resected spinal accessory nerve.

 The removal of the internal jugular vein has minimal impact on the quality of life for head and neck cancer patients. Postoperative edema is a transitory condition that usually disappears in a week time. The collateral veins of the neck prevent the occurrence of this situation (**Fig. 3**). Despite this favorable situation, there is the need of limiting excessive hydration of the patient because of the possibility of inappropriate antidiuretic hormone secretion (ADH). The excessive ADH secretion, which in not uncommon in head and neck cancer patients, can lead to the accumulation of fluids in soft tissues. Persistent edema after radical neck dissections is unlikely to occur. Still, the removal of both internal jugular veins in the same surgical intervention is not to be performed because of the risk of having significant edema in the head and neck region that can be fatal for the patient. When needed, bilateral internal jugular vein resection, is to be done on a second stage procedure at least 6 weeks apart. This spacing allows the formation or the recalibration of collateral veins. When operating a patient with bilateral neck metastasis the tumor is to be resected in the second stage procedure along with the remaining contralateral neck metastasis. This follows the principle of resecting the tumor and the neck metastasis all together.

## Conclusions

Radical neck dissection and modified neck dissection have demonstrated their efficacy in controlling tumor metastasis in head and neck region in patients with early stage cancer. When performing radical neck dissection the increased mortality and morbidity for patients in early stages lead us to use a more conservative neck dissection such as the modified neck dissection. The surgical indications for the performing modified neck dissection are early stage metastatic disease, patients without multiple cervical metastases at different sites and the absence of the bulky disease.

 On the other hand, the radical neck dissection still finds its use when dealing with advanced and extended tumor process in the head and neck area towards the skin, cervical vessels (carotid arteries), nerve or deep tissues spread. The life of the patient is the main concern of the surgeon when dealing with such cases. 

 The quality of life is a second hand issue when the tumor process is extremely advanced. If an oncological procedure is not possible, the surgeon needs to take into consideration the possibility of a palliative surgical intervention that allows the patient to live a decent life and have some quality of life. Nevertheless, feeding is one of the most important things for a late stage cancer patient, thus making it necessary for the surgeon to make sure that the patient has the possibility of feeding. Advanced pharynx and larynx cancer patients along with thyroid cancer patients have an impaired deglutition. If the surgical procedure does not assure the digestive system continuity, the patient will have to undergo a gastrostomy or a jejunostomy for enteral feeding. This scenario usually occurs in the recurrence of the neck disease.

 When possible, the preservation of the spinal accessory nerve is a technique that helps decrease morbidity and prevents shoulder dysfunction as well as the absence of pain where the ligation and resection of the internal jugular vein is linked to facial swelling, and an increase in the pressure level inside the cranium when performed bilaterally. This is avoided by the two-stage surgery.

 The quality of life of head and neck patients is in direct correlation with the cancer stage they present themselves to the physician. Late stages may lead to extensive oncological surgery, which may cause the appearance of deformities in the head and neck region and dysfunctions.

 Morbidity and mortality are decreased by assessing data about the clinical status of the patient, evidence-based decisions according to therapy guidelines and adequate surgery indications. We should not abandon the oncological principles in favor of the esthetics and functionality. This is only possible if the patient is well informed about the surgery and the supposed outcome and the patient agrees on having the oncological therapy.
